# Synaptic Actions of Amyotrophic Lateral Sclerosis-Associated G85R-SOD1 in the Squid Giant Synapse

**DOI:** 10.1523/ENEURO.0369-19.2020

**Published:** 2020-04-09

**Authors:** Yuyu Song

**Affiliations:** 1 Department of Genetics, Yale School of Medicine, Howard Hughes Medical Institute, New Haven, CT 06510; 2 Laboratory of Systems Pharmacology, Program in Therapeutic Science, Harvard Medical School, Boston, MA 02115; 3 Department of Neurology, Massachusetts General Hospital, Charlestown, MA 02129; 4 Marine Biological Laboratory, Woods Hole, MA 02543

**Keywords:** amyotrophic lateral sclerosis associated, calcium, giant synapse, neurodegeneration, SOD1, synaptic vesicles

## Abstract

Altered synaptic function is thought to play a role in many neurodegenerative diseases, but little is known about the underlying mechanisms for synaptic dysfunction. The squid giant synapse (SGS) is a classical model for studying synaptic electrophysiology and ultrastructure, as well as molecular mechanisms of neurotransmission. Here, we conduct a multidisciplinary study of synaptic actions of misfolded human G85R-SOD1 causing familial amyotrophic lateral sclerosis (ALS). G85R-SOD1, but not WT-SOD1, inhibited synaptic transmission, altered presynaptic ultrastructure, and reduced both the size of the readily releasable pool (RRP) of synaptic vesicles and mobility from the reserved pool (RP) to the RRP. Unexpectedly, intermittent high-frequency stimulation (iHFS) blocked inhibitory effects of G85R-SOD1 on synaptic transmission, suggesting aberrant Ca^2+^ signaling may underlie G85R-SOD1 toxicity. Ratiometric Ca^2+^ imaging showed significantly increased presynaptic Ca^2+^ induced by G85R-SOD1 that preceded synaptic dysfunction. Chelating Ca^2+^ using EGTA prevented synaptic inhibition by G85R-SOD1, confirming the role of aberrant Ca^2+^ in mediating G85R-SOD1 toxicity. These results extended earlier findings in mammalian motor neurons and advanced our understanding by providing possible molecular mechanisms and therapeutic targets for synaptic dysfunctions in ALS as well as a unique model for further studies.

## Significance Statement

The squid giant synapse (SGS) presents one of the few mature nervous systems *in situ* that mimics mammalian neuromuscular junctions, while allowing precise experimental manipulations and live measurement with superior spatial and temporal resolution. Applying these unique features to studying the molecular mechanisms of amyotrophic lateral sclerosis (ALS), a devastating adult-onset neurodegenerative disease without cure, offers clues to understand the pathogenesis of the disease. Our results demonstrating synaptic dysfunction caused by ALS-associated mutant SOD1 protein and its underlying molecular pathways may suggest a novel approach to an effective therapeutic intervention as well as identify biomarkers for early diagnosis. Furthermore, the altered synaptic vesicle behavior and Ca^2+^ dynamics revealed through the perturbation of neurotransmission by ALS extends our understanding of fundamental synaptic physiology at both molecular and cellular levels.

## Introduction

Amyotrophic lateral sclerosis (ALS) is a fatal adult-onset neuromuscular disease with dysfunction and loss in both upper and lower motor neurons, resulting in progressive muscle weakness and atrophy, eventual paralysis, and death usually within three to five years after initial diagnosis. Inherited forms of ALS represent roughly 10% of cases, with similar clinical symptoms to sporadic ALS patients. Unfortunately, there is still no effective cure for ALS. To enable therapeutic intervention at an early stage of the disease, the mechanisms underlying ALS and presymptomatic biomarkers for early diagnosis must be identified.

Mutations in Cu/Zn superoxide dismutase (SOD1) are responsible for ∼25% of familial ALS cases, but SOD1 is expressed in many cell types where it removes superoxide radicals and is a component of redox signaling pathways. ALS-associated mutations induce a conformational change within SOD1 due to protein misfolding (Bruijn et al., 1998) and aberrant interactions with other proteins (Pasinelli et al., 2004; Urushitani et al., 2006). Genetic knock-out of SOD1 does not produce motor deficits seen in ALS, indicating that SOD1 mutations confer a toxic gain-of-function (Bunton-Stasyshyn et al., 2015; Taylor et al., 2016; Ghasemi and Brown, 2018). A glycine-to-arginine substitution at residue 85 (G85R) is one highly studied ALS-associated mutation: G85R-SOD1 is incapable of dimerizing normally and instead misfolds into soluble monomers or oligomers, as well as insoluble aggregates that are toxic to motor neurons (Wang et al., 2009a,b).

To characterize G85R-SOD1 toxicity specific to neurons, various forms of misfolded G85R-SOD1-YFP were perfused into isolated axoplasm from squid giant axons (Song et al., 2013), and both soluble monomers and oligomers inhibited axonal transport from cell body to synapse through aberrant activation of signaling pathways (Song et al., 2013). Although G85R-SOD1 inhibition of anterograde transport of synaptic components could lead to synaptic dysfunction, questions remained as to whether G85R-SOD1 has a direct effect on synaptic transmission. Impairment of neuromuscular junctions is detected early in patients and animal models (Moloney et al., 2014), consistent with a “dying-back” neuropathy where synaptic dysfunction and loss is a primary pathology eventually leading to neuronal death. Decreased synaptic vesicle numbers and reduced presynaptic proteins in presynaptic terminals of several ALS animal models further suggest a presynaptic effect (Wang et al., 2009a). These changes are seen before motor deficits are detectable, suggesting that synapses could be a primary target of G85R-SOD1 (Frey et al., 2000; Fischer et al., 2004; Hegedus et al., 2007; Sakowski et al., 2012).

Synaptic transmission is altered in ALS mouse models and induced Pluripotent Stem Cells (iPSCs)-derived motor neurons, raising key questions: how do ALS-associated proteins alter synaptic functions? Can synaptic function be restored? Unfortunately, the complexity of mouse motor systems, chronic nature of disease pathology, small size of presynaptic domains, and difficulty of targeting synapses limit utility of mouse and cell culture models in addressing questions about presynaptic mechanisms.

The squid giant synapse (SGS), which mimics neuromuscular junctions with similar ion channel compositions and neurotransmission machinery (Katz and Miledi, 1967, 1977), was used to evaluate presynaptic and postsynaptic actions of G85R-SOD1 here. The large size of squid giant presynaptic terminals permits experimental manipulations such as protein microinjection, direct electrophysiological measurements of transmission, current and voltage clamping, and Ca^2+^ imaging, facilitating detailed studies of presynaptic molecular mechanisms (Bloedel et al., 1966; Llinas et al., 1980, 1994; Augustine and Charlton, 1986; Llinás et al., 1985; Augustine et al., 1988, 2006; Augustine, 1990; Lin et al., 1990; Fukuda et al., 1995; Sugimori et al., 1998). The squid synapse is particularly suitable for studying ALS, because it is (1) fast-transmitting with high release capacity, similar to synapses affected in ALS; and (2) enriched in ion channels (e.g., Ca^2+^), neurotransmitters (e.g., glutamate) and receptors (e.g., AMPAR) implicated in ALS pathology.

Here, we microinjected G85R-SOD1 in presynaptic terminals, recorded both presynaptic and postsynaptic membrane potentials with and without high-frequency stimulations (HFSs), performed live ratiometric Ca^2+^ imaging, and examined synaptic vesicle morphology/distribution by electron microscopy (EM). Correlated functional and structural studies demonstrated the acute synaptic inhibition by G85R-SOD1 as evidenced by diminished postsynaptic potentials (PSPs), decreased synaptic vesicle number at active zones (AZs), and reduced synaptic mobilization from reserved (RP) to readily releasable pool (RRP) within 30 min after G85R-SOD1 injection. Unexpectedly, while continuous trains of HFS depleted synaptic vesicle availability and enhanced synaptic inhibition by G85R-SOD1, intermittent HFS (iHFS) applied every 30 min before and during G85R-SOD1 infusion kept synapses firing normally for 7 h. Given the effects of HFS on presynaptic Ca^2+^, we tested for aberrant Ca^2+^ signaling by ratiometric Ca^2+^ imaging with presynaptic fura-2. Ca^2+^ increased throughout terminals including in “palm” regions before presynaptic axons branch, which lack plasmalemmal Ca^2+^ channels. Reducing intracellular Ca^2+^ by EGTA rescued synaptic function and restored normal structure in the presence of G85R-SOD1 as did iHFS. These results are consistent with and extend findings in mammalian nervous systems where neurons with lower Ca^2+^ buffering capacity are selectively affected in ALS and these neurons showed Ca^2+^ imbalances presymptomatically (Grosskreutz et al., 2010). Understanding sources of increased Ca^2+^ and molecular mechanisms underlying Ca^2+^ activation as well as how iHFS rescues synaptic function will provide unique insights into synaptic dynamics and a possible molecular basis for synaptic pathology in human ALS patients.

## Materials and Methods

### Proteins and reagents

G85R-SOD1-His, WT-SOD1-His, G85R-SOD1-His-YFP, and WT-SOD1-His-YFP were produced in *E. coli* BL21/DE3 cells under pET vectors, and purified on Talon resin first and then by chromatography on MonoQ 10/10, in the Horwich Laboratory as described previously (Song et al., 2013). All chemicals used were American Chemical Society quality or better, from Sigma and Invitrogen (now Fisher Scientific). For microinjection, proteins and reagents were dissolved in 200 mM KCl, 100 mM taurine, 250 mM K-isethionate, and 50 mM K-HEPES, pH 7.4). Tetramethylrhodamine-dextran (3 kDa, Invitrogen) was used for co-injection with non-fluorescent reagents to visualize and monitor the injection.

### SGS preparation and electrophysiology setup

The giant squid synapse is formed between the terminal finger of second order axons of the pre-nerve and the third order axon of the last stellate nerve, which is the giant axon used for axonal transport studies as well as for classical voltage-clamp studies of ion channels (Young and Keynes, 2005). Following a standard protocol (Llinas et al., 1980, 1981, 1994; Llinás et al., 1985; Augustine and Charlton, 1986; Augustine et al., 1988, 2006; Augustine, 1990; Smith et al., 1993), stellate ganglia of small female *Loligo pealeii* (RRID: SCR_002864, Marine Biological Laboratory) were removed from the mantle carefully and rapidly under running seawater, tied off at each end of the presynaptic and postsynaptic axons, isolated from the sheath and connective tissues, pinned with fine cactus needles to a thin-layer of Sylgard on the bottom of a 35-mm Petri dish chamber, superfused continuously with oxygenated squid saline (455 mM NaCl, 54 mM MgCl_2_, 11 mM CaCl_2_, 10 mM KCl, 3 mM NaHCO_3_, and 10 mM HEPES, pH 7.2) at 10–15°C.

**Figure 1.**
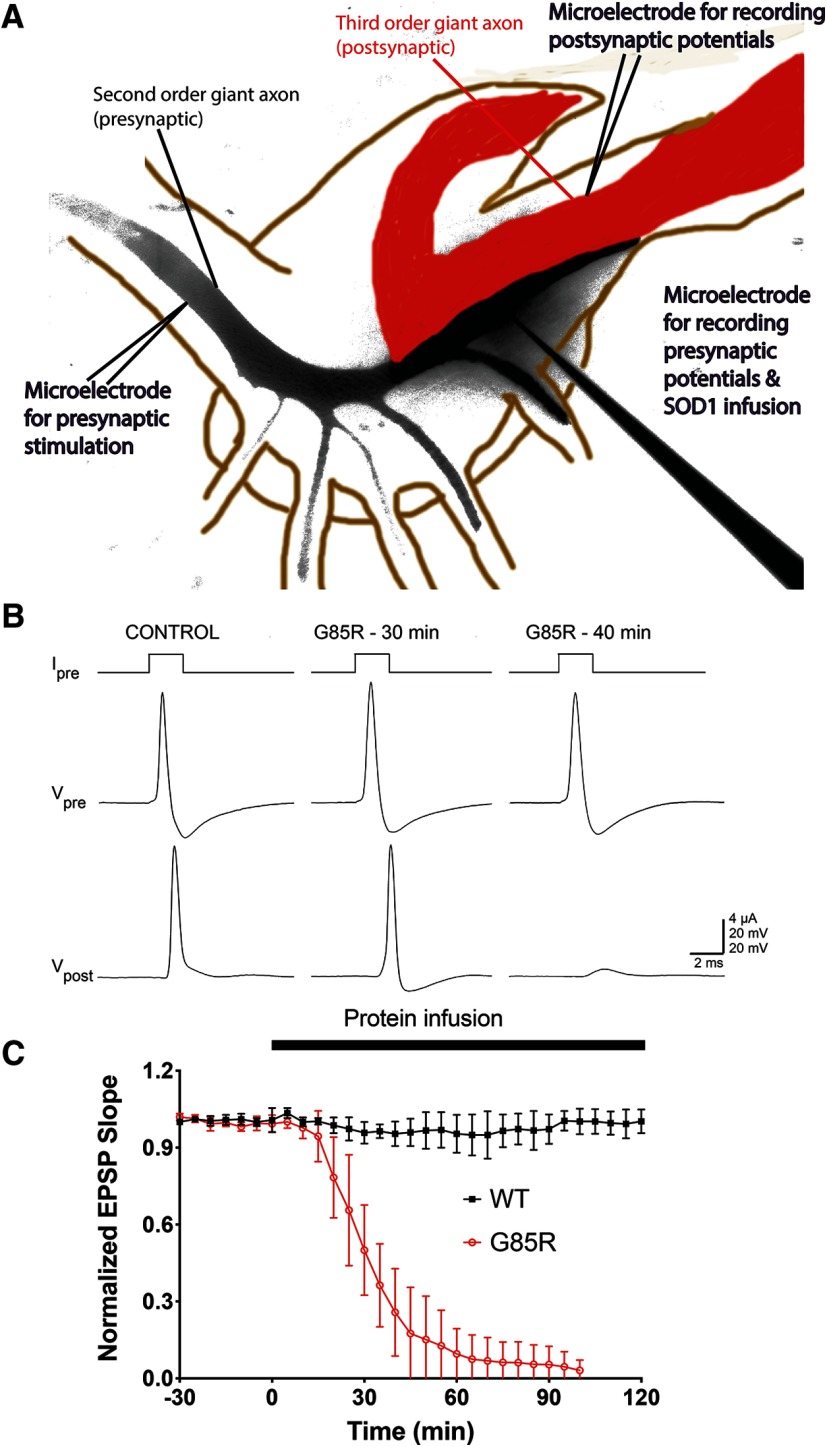
Presynaptic infusion of G85R-SOD1-YFP inhibited synaptic transmission. ***A***, Diagram of experimental setup. Presynaptic second order giant axon branch (black) and postsynaptic third order giant axon (red) form the giant synapse in the stellate ganglion. Two electrodes were inserted in the presynaptic axon, one at the palm to deliver electronic stimulation at 0.03 Hz (each pulse is 2 μA for 2 ms) and the other in the presynaptic terminal for recording presynaptic potentials as well as injecting 50 μM SOD1 proteins and reagents of interest at 0.1 Hz, each injection was 50 psi for 250 ms. The third electrode was inserted in the postsynaptic axon to record PSPs. ***B***, Under current clamping condition, G85R-SOD1-YFP-injected synapses showed reductions in PSP (V_post_) within 30 min and a failure to elicit a postsynaptic action potential in 40 min, compared with the control before injection. ***C***, EPSP slopes of WT-SOD1-YFP-injected synapses (*n* = 5) and G85R-SOD1-YFP-injected synapses (*n* = 8) were normalized to the initial time point (−30 min), 30 min before the beginning of SOD1 injections. Averaged EPSP slopes were plotted with error bars showing standard error (SE). The thick black bar indicates the duration of SOD1 infusion starting at time 0. G85R-SOD1-YFP consistently inhibited synaptic transmission as shown by reductions in the EPSP slope, while WT-SOD1-YFP infusions showed no effect on EPSPs. Control synapses infused with fluorescent dextran showed normal firing similar to the WT-SOD1-YFP-infused synapses (Extended Data Fig. 1-1). Moreover, synaptic function gradually recovered in G85R-SOD1-YFP-infused synapses after protein infusion was stopped, excluding the possibility of physical damage of synaptic machinery due to injection (Extended Data Fig. 1-2).

The most distal digit of the presynaptic axons and the most medial fiber, also known as the giant axon, form the giant synapse (Fig. 1*A*). One microelectrode for injecting was inserted in the presynaptic axon to inject current at 0.033 Hz for basal stimulation and at 50 Hz for HFS (each pulse is 2 μA for 2 ms for basal stimulation and 2 μA for 1 ms for HFS), near the palm where the second order axon enters the ganglion to branch and form multiple synapses with postsynaptic axons. At the terminal of the most medial branch, a second microelectrode was inserted to presynaptically inject proteins and reagents of interest as well as recording the presynaptic membrane potentials. Finally, at the postsynaptic terminal of the giant synapse, a third microelectrode was inserted near the medial presynaptic digit to record the postsynaptic membrane potentials. Electrodes 1 and 3 were filled with 3 M KCl while the second microelectrode allowed the injection of 50 μM SOD1 proteins or reagents of interest in 100 mM KCl (Smith et al., 1993) at 0.1 Hz (each injection was 50 psi for 250 ms). The injection efficiency of SOD1 proteins was monitored by a fluorescence microscope. All synapses were injected with the same pressure for the same amount of time during data acquisition, and the final mean fluorescence intensities in the presynaptic terminals were measured and compared between samples to ensure comparable levels of infused proteins.

10.1523/ENEURO.0369-19.2020.f1-1 Extended Data Figure 1-1. No effects of fluorescent dextran on synaptic transmission. Synapses infused with rhodamine-dextran alone kept firing for >2 h, without significant changes in synaptic strength as evidenced by constant postsynaptic membrane potential waveform (***A***) and EPSP slope (***B***). Download Figure 1-1, TIF file.

**Figure 2:**
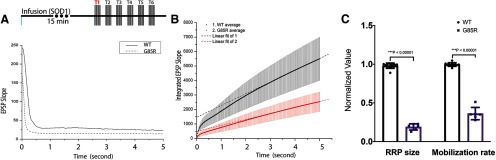
Presynaptic infusion of G85R-SOD1-YFP altered synaptic vesicle dynamics. ***A***, Series of six continuous trains of HFS (each train is 50 Hz for 5 s with 5 s between trains) was applied to the synapses infused with SOD1 proteins for 15 min before HFS trains. WT-SOD1-GFP-injected synapses showed constant EPSP slope at the beginning of each train suggesting robust neurotransmission (>200, also see Extended Data Fig. 2-1 for the sixth train). However, G85R-SOD1-YFP-injected synapse started to show reductions in synaptic transmission, as evidenced by the dramatic decrease in EPSP slopes taken during the first train both at the beginning and at the steady state, suggesting severe depletion of synaptic vesicles from both the RRPs and the RPs. This inhibition was more obvious in the sixth train (Extended Data Fig. 2-1). ***B***, EPSPs of the first train from WT-SOD1-YFP-injected synapses (black, *n *= 11) and from G85R-SOD1-YFP-injected synapses (red, *n* = 6) were integrated and averaged, followed by linear fit. The intersection with *y*-axis indicated the size of the RRP and the slope indicated the mobilization rate of vesicles from the RP to RRP. ***C***, Normalized to baseline values before the injection of SOD1-YFP, RRP size and mobilization rate from the first train were plotted as individual biological replicates (*n =* 11 for WT-SOD1-infused synapses and *n =* 6 for G85R-SOD1-infused synapses) to show significant reductions in both RRP and mobilization of vesicles from the RP by G85R-SOD1-YFP, but not by WT-SOD1-YFP.

10.1523/ENEURO.0369-19.2020.f1-2 Extended Data Figure 1-2 Recovery of synaptic transmission after the removal of G85R-SOD1-YFP. The synapse was injected with G85R-SOD1-YFP continuously for 30 min until the significant reduction in EPSP slope and the failure to elicit a postsynaptic action potential. As the injection stopped, G85R-SOD1-YFP slowly diffused away from the presynaptic terminal to the axon, leading to a full recovery of EPSP after 150 min, suggesting that synaptic machinery remained intact after treatment. After the synapse and neurotransmission were stabilized for more than 1 h, G85R-SOD1-YFP was injected again and a similar inhibitory effect was observed. Download Figure 1-2, TIF file.

### Electrophysiology data acquisition and analysis

Both presynaptic potential and PSP were recorded using sharp microelectrodes with an Axoclamp-2A amplifier (Axon Instrument) and data analyzed using Labview (National Instruments) software (Yulong Li). Raw waveforms were used to calculate PSP slope using the same parameters for all experiments. For data acquired under HFS, PSP slopes were analyzed, integrated, plotted, and fitted linearly using the last 50 time points to derive the vesicle mobilization rate (slope of the linear fit) and the RRP size (intersection with the *y*-axis).

### Ratiometric Ca^2+^ imaging

We dissolved 1 mM fura-2 (pentapotassium salt, ThermoFisher F1200, CHEBI:52 081), a Ca^2+^ indicator, in 100 mM KCl and infused it into the presynaptic terminal by electrophoresis (100 nA current) through the first presynaptic electrode inserted in the palm. Fura-2 infusion continued for 10 min, and the synapse was allowed to equilibrate for 30 min before presynaptic injection of SOD1 proteins through the second microelectrode to ensure fura-2 was diffused evenly throughout the whole presynaptic terminal at roughly 100 μM. Ratiometric live Ca^2+^ imaging was performed every 30 s with ratiometric images taken at Ex360 nm and Ex390 nm assisted by an ultra-high-speed wavelength switching λ DG-4/5 xenon arc lamp system. Ratios at various locations (A) were calculated as (fluorescent intensity^360nm^)/(fluorescent intensity^390nm^) as a measure of intracellular Ca^2+^ concentration (Smith et al., 1993). In vitro calibration was performed by mixing squid axoplasm with equal volumes of buffer containing 5 μM fura-2, 400 mM KCl, and 40 mM Na-HEPES (pH 7.2). Three doses of CaCl_2_ were set by adding 20 mM EGTA (low Ca^2+^, <10^−8^ M), or 20 mM EGTA + 13.3 mM CaCl_2_ (intermediate Ca^2+^, ∼6.7 × 10^−7^ M), or 20 mm CaCl_2_ (high Ca^2+^). Various ratios under each condition were measured using the same optics and the background noise was subtracted. The dose response curve was obtained to allow the determination of Kd′ and the two ratios. Rmax was defined as the ratio value at saturating Ca^2+^ level, and Rmin was defined as the ratio value at the minimal Ca^2+^ concentration.

### EM

After recordings, synapses were fixed in 4% glutaraldehyde and 2% paraformaldehyde in 0.1 M cacodylate buffer for 12 h at 4°C, then washed in 0.1 M cacodylate buffer three times and postfixed in 1% osmium tetroxide for 2 h at 4°C, followed by blocking impregnation with 2% uranyl acetate in 0.1 M sodium acetate, pH 5.0 for 24 h. After washing, dehydration was performed in ethanol with increasing concentration: 50% for 10 min, 75% for 10 min, 80% for 10 min, 85% for 10 min, 95% for 10 min, and 100% for 5 min twice, followed by propylene oxide for 5 min three times. Resin and propylene (1:1 for 24 h, 2:1 overnight, and 3:1 for 2–4 h, and finally 100% resin overnight) infiltration was conducted before embedding in silicone molds in the oven at 62°C for 72 h. Sectioning and imaging were performed following standard EM protocols (Augustine et al., 2006; Moreno et al., 2009). Vesicle density at the active zones (AZs) was determined as the number of vesicles per square micrometer. Distance between vesicles and AZs was measured and plotted as described previously (Morgan et al., 1999). All EM samples were numbered, processed, and analyzed under blinded conditions until the last step when data had to be pooled.

### Statistical information

All experiments were repeated at least five times. The data were analyzed by one-way ANOVA followed by the Tukey *post hoc* test (or nonparametric multiple *t* tests, without assuming consistent SD) and plotted in Prism 7 (GraphPad software). Quantitative data were plotted as mean ± SEM, *p* values were calculated and four statistical thresholds were marked: *p* < 0.00,001, *p* < 0.0001, *p* < 0.001, and *p* < 0.005 (*p* < 0.05 indicated statistical significance). For EM analysis, nested one-way ANOVA was performed to compare WT and G85R-infused synapses (102 synapses across three synapses for each group).

## Results

### G85R-SOD1-YFP but not WT-SOD1-YFP inhibits synaptic transmission

To assess whether the mutant SOD1 is directly toxic to the synapse, as opposed to indirectly affecting it, e.g., by blocking axonal anterograde vesicular trafficking (Song et al., 2013), G85R-SOD1-YFP (*n* = 8) or WT-SOD1-YFP control (*n* = 5) was microinjected in the presynaptic terminal site (Fig. 1*A*) through the micropipette extending in from lower right; note that black color indicates the extent of YFP fluorescence after 40 min of infusion. Another microelectrode was used to pass current pulses for basal stimulation (2 μA × 2 ms, every 33 s; Fig. 1*A*, micropipette extending in from lower left) to evoke single action potentials, detected by intracellular voltage recording simultaneously from both presynaptic and postsynaptic sites (Fig. 1*A* for sites of recording and Fig. 1*B*, middle and bottom traces). This protocol is referred to hereafter as “basal stimulation.” Measurement of voltage changes at both presynaptic and postsynaptic sites allows for assessment of the efficiency of synaptic transmission. Strikingly, after 40 min of G85R-SOD1-YFP infusion, the postsynaptic action potential was no longer detected, with only a subthreshold PSP observable (Fig. 1*B*, Vpost). By comparison, there was no change in the PSP with a WT-SOD1-YFP infusion (Fig. 1*B*, control) or with fluorescent dextran alone (Extended Data Fig. 1-1). A time course study of WT-SOD1-YFP and G85R-SOD1-YFP was conducted to observe the kinetic behavior of EPSPs from basal stimulation during the respective infusions (Fig. 1*C*; note that points are shown only for every 10th pulse, i.e., at 5-min intervals). With infusion of the mutant protein, the EPSP slope began to reduce by ∼10 min, and was completely lost by ∼60–70 min. By contrast, no effect on EPSP was observed with WT-SOD1-YFP infusion for >100 min (Fig. 1*C*). Thus, in contrast with the absence of an observable effect of WT-SOD1 infusion on postsynaptic EPSP, injection of monomeric G85R-SOD1-YFP produced a substantial time-dependent inhibition of synaptic transmission.

To rule out the possibility that synaptic defects were caused by physical damage induced by electrode impalement and microinjection, injection was stopped when postsynaptic action potentials were abolished. This allowed the diffusion of G85R-SOD1-YFP away from the synaptic terminal through the presynaptic axon. The EPSP slope started to increase 90 min later as the presynaptic fluorescent intensity of G85R-SOD1-YFP significantly decreased and EPSPs were fully restored in ∼2.5 h after most mutant SOD1 had diffused out of the terminal (Extended Data Fig. 1-2). Normal synaptic transmission continued for >1 h until a second injection of G85R-SOD1-YFP significantly inhibited synaptic transmission in the same fashion as the first injection. These results suggested that G85R-SOD1-YFP inhibits synaptic transmission at least in part by directly affecting neurotransmitter release from the synaptic terminal and this effect was reversible on the removal of G85R-SOD1-YFP from the synaptic terminal.

### G85R-SOD1-YFP impairs synaptic vesicle availability at the AZ

To examine whether G85R-SOD1-YFP inhibited synaptic transmission by affecting synaptic vesicle pool dynamics, we microinjected G85R-SOD1-YFP or WT-SOD1-YFP for 15 min and then applied trains of HFS presynaptically (50 Hz for 5 s, with 5 s intervals between trains) and measured postsynaptic depression of EPSPs, which are proportional to the underlying EPSCs and are uncontaminated by action potentials, to determine the size of the RRP and the rate of vesicle mobilization from the reserved pool (RP). EPSP slopes during both the first train of a series trains of HFS (Fig. 2*A*) and the sixth train (Extended Data Fig. 2-1) were compared in WT and G85R synapses. For controls, there was a two-phase diminution of EPSP slope across the 250 spikes, whereas with G85R synapses the first phase was barely detectable during the sixth train of HFS and was significantly inhibited during the first train. The first phase is generally associated with utilization of the RRP, which appears to be smaller with the mutant, whereas the second phase is generally associated with utilization of the RP, which appears to be present in both with significant reduction by G85R-SOD1-YFP. To further measure these pools, the EPSP slope values were continuously integrated across one train of the same synapse before infusion and one train after 30 min of WT- or G85R-SOD1-YFP infusion. The EPSPs of each condition were integrated and averaged to generate traces (Fig. 2*B*). Notably, the WT-SOD1 trace resembled that of the preinfusion trace (not shown), indicating that WT-SOD1 has little effect on synaptic vesicle availability and mobilization, whereas G85R-SOD1 trace exhibited integrated values that were substantially lower than those of WT-SOD1. The relative size of the RRP was determined for each condition by intersection of slope with the *y*-axis, and the mobilization rate from the RP was directly determined from the slopes (dotted lines; linear fit of the integrated EPSP slopes from the last 50 time points). The values and statistics for independent experiments are shown (*n* = 11 for WT and *n* = 6 for G85R; Fig. 2*C*). G85R-SOD1-YFP drastically reduced both the RRP size and the vesicle mobilization rate from RP to RRP as compared with WT-SOD1-YFP. Therefore, reduced RRP size and slower RP mobilization rate, could lead to the loss of synaptic transmission.

10.1523/ENEURO.0369-19.2020.f2-1 Extended Data Figure 2-1 Significant inhibition of EPSP by continuous HFS in G85R-SOD1-YFP-infused synapse. EPSP slopes taken during the 6th train in a set of continuous trains of HFS showed even more dramatic decreases by G85R-SOD1-YFP, compared with the WT-SOD1-YFP, particularly at the beginning, suggesting limited synaptic vesicle availability, consistent with the morphological changes found in EM. Note that in panel ***A***, the EPSP slope with G85R-SOD1-YFP in the 1st train was initially >150, while in the 6th train, it never got above 40. In contrast, the initial EPSP slopes in the 1st train and the 6th train were almost identical after perfusion with WT-SOD1-YFP. Download Figure 2-1, TIF file.

### EM of WT-SOD1-YFP and G85R-SOD1-YFP-infused synapses

**Figure 3.**
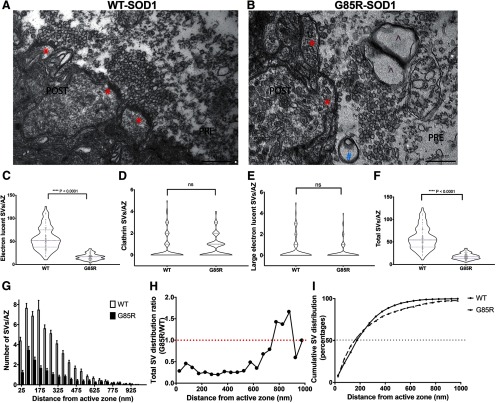
Presynaptic infusion of G85R-SOD1 inhibited synaptic vesicle (SV) availability. Representative EM images illustrate morphology of AZs (labeled with red *) and numbers of SVs in fixed synapses infused with WT-SOD1 (***A***, *n* = 3) and G85R-SOD1 (***B***, *n* = 3). G85R-SOD1-infused synapses showed vacant AZs and occasional abnormal membranous structures (indicated by blue # and purple ^⋀^). ***C–F***, Quantification of averaged vesicle number across all AZs from 102 WT- and 102 G85R-SOD1-YFP-infused synapses showed statistically significant reductions in the total vesicle number and in the electron lucid vesicle number by G85R-SOD1-YFP as compared with WT-SOD1. The clathrin-coated vesicles and the large electron lucid vesicles were comparable between WT- and G85R-SOD1-YFP-infused synapses. Nested one-way ANOVA was performed to compare WT and G85R-infused synapses (*p* < 0.0001) as well as across biological triplicates within each group (*p* > 0.05) ns: not significant. ***G***, Averaged number of SVs per AZ were plotted along the distance from AZ (binned by 50 nm). ***H***, Distance distribution plot of SVs in each 50-μm bin showed a global reduction of SVs from G85R-SOD1-YFP-infused synapses regardless of the distance from the AZs. Because of the drastically reduced numbers of SVs far (>675 nM) from the AZs in both WT and G85R synapses, the reduction seemed to disappear or even be reversed, however, the differences far from the AZ may not be significant due to the substantially decreased numbers of vesicles in that area for both WT and G85R synapses. ***I***, Cumulative SV distribution plots showed similar distribution patterns of vesicles in WT (solid line) and G85R (dashed line) synapses, confirming the even inhibition of SV availability by G85R-SOD1 independent of the distance from the AZs.

To visualize the synaptic vesicles directly, we conducted EM analyses of synapses infused for 40 min under basal stimulation with the respective proteins (*n* = 3 for each group). The WT-SOD1-YFP-infused synapses exhibited normal morphology with abundant packing of synaptic vesicles against the presynaptic membrane, comparable to published EM data (Augustine et al., 2006) of normal SGS (Fig. 3*A*, red * indicates an AZ), whereas the G85R-SOD1-YFP-infused synapses reproducibly exhibited synaptic vesicles that did not abut the membrane at AZs, lying in a zone distant from it (Fig. 3*B*). The vesicles present in this zone also appeared generally larger, less compact in morphology and reduced in number relative to WT-SOD1 synapses. We also consistently observed additional structures, e.g., in this image, what appears to be a pre-autophagosome (labeled by blue #) and two adjoining prelysosomes (labeled by purple ^). By contrast we did not observe such structures in WT-SOD1-infused presynaptic AZs. Notably, many of the pre-autophagosomes appeared to contain vesicles. This potentially reflects a local mechanism to remove G85R-SOD1-YFP-affected synaptic vesicles in the RRP and a subpopulation of vesicles in the RP, whereas, interestingly, the most remote RP pools appear to be spared, suggesting a potential source for synaptic vesicle supply, which may help restoring synaptic function if released on proper stimulations. More detailed characterization of affected vesicles was conducted by counting the number of electron lucent vesicles, clathrin-coated vesicles, and large electron lucent vesicles per AZ as described previously (Morgan et al., 1999; Fig. 3*C–F*). Nested ANOVAs suggested that while clathrin-coated vesicles did not seem to be affected by G85R-SOD1, the total number of available synaptic vesicles was dramatically reduced. A cumulative measurement of AZs (102 across three biological repeats per group) infused with WT-SOD1 or G85R-SOD1 (binned by 50 μm in distance) showed that such reductions seem to be evenly distributed in the RRP regardless of the distance from the membrane (Fig. 3*G–I*). Because of the limited number of vesicles further away from the AZs (>675 μm) in both groups, the increase in the relative vesicle distribution in that vicinity may not be statistically different between the G85R-SOD1-infused synapses and the WT-SOD1-infused synapses. This is further confirmed by the cumulative vesicle distribution curves (without binning) in WT and G85R-SOD1 synapses (Fig. 3*I*).

### Prevention of G85R-SOD1-YFP-associated synaptic deficits by iHFS

**Figure 4.**
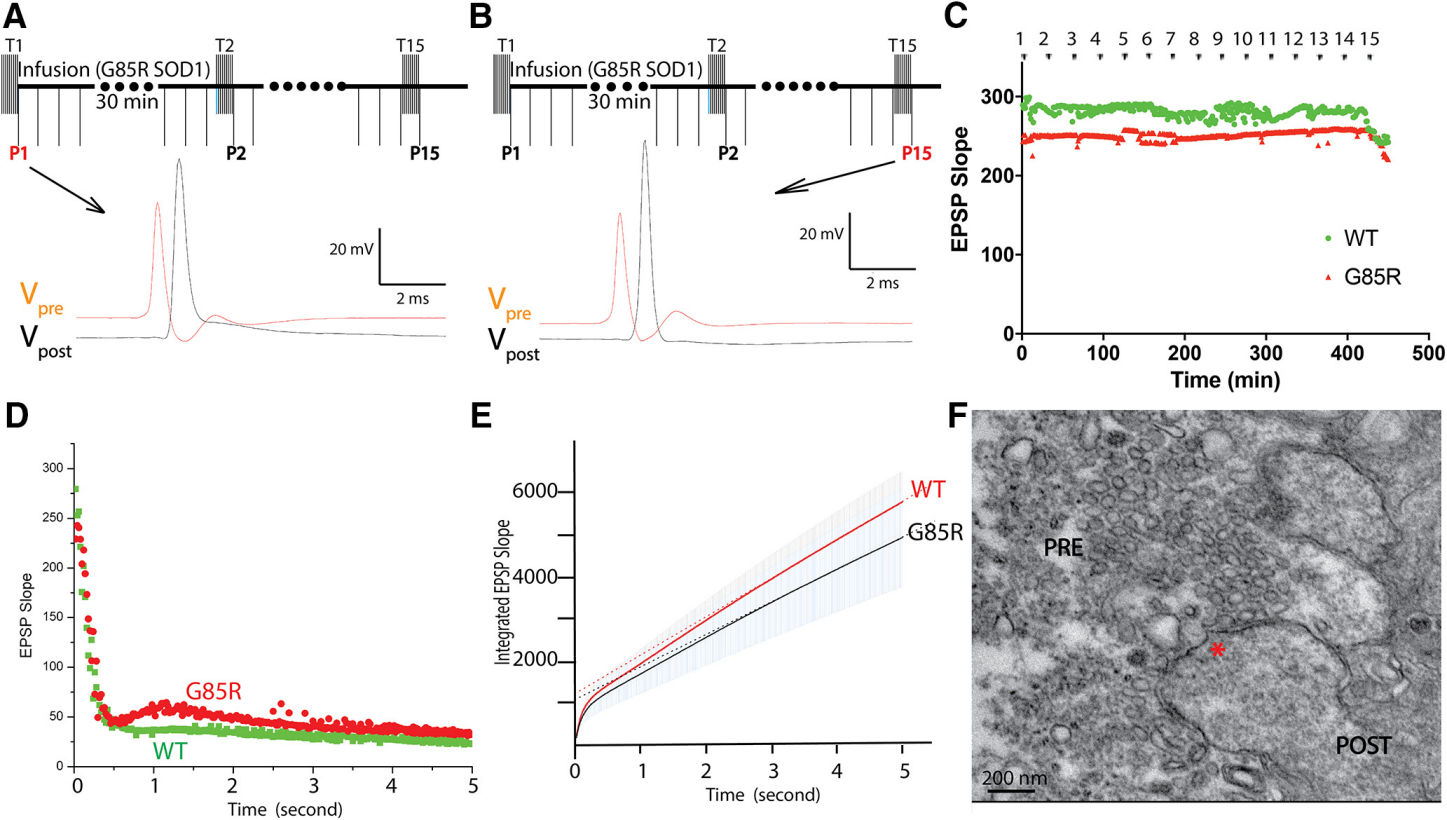
iHFS prevented synaptic transmission deficits caused by G85R-SOD1-YFP. ***A***, iHFS (5 s of 50 Hz applied every 30 min) was first applied to the synapse at time 0 before G85R injection, both presynaptic and postsynaptic membrane potentials were recorded 1 min after HFS (P1). ***B***, If iHFS trains were applied every 30 min before and during continuous SOD1-YFP infusion into the synapse, G85R-SOD1 no longer inhibited synaptic transmission and the synapse continued to fire even after 7 h without significant changes in either presynaptic or postsynaptic membrane potentials (P15). ***C***, EPSP slopes remained constant and comparable under basal stimulation with iHFS in WT and G85R synapses. ***D***, EPSP slope from one single train of iHFS showed no significant difference between WT and G85R synapses except for occasional augmentation seen in G85R-SOD1-YFP-infused synapses. ***E***, To evaluate the vesicle dynamics, EPSP slopes were measured and integrated during HFS trains applied either with 2 h WT- or G85R-SOD1-YFP infusion in the presence of iHFS every 30 min (*n* = 6). There were no significant differences in RRP size and mobilization rate of vesicles trafficking from RP to RRP by G85R-SOD1-YFP. ***F***, EM showed normal presynaptic structure with normal numbers of vesicles at the AZ (indicated by red asterisk) in G85R-SOD1-YFP-infused synapses when iHFS was applied. Interestingly, in three out of five synapses, HFS restored firing in G85R-SOD1-YFP-infused synapses, where EPSPs were significantly inhibited (Extended Data Fig. 4-1).

Unexpectedly, when a single train of HFS (50 Hz for 5 s) was applied presynaptically every 30 min, G85R-SOD1-YFP-infused synapses, otherwise receiving continuous basal stimulation, maintained constant EPSPs for over 8 h (Fig. 4*A–C*). This contrasts with the finding (Fig. 1) that in the setting of G85R-SOD1-YFP infusion, continuous basal stimulation leads to steady decline and then complete loss of EPSPs within 40–60 min. This also contrasts with the finding (Fig. 2) that continuous HFS trains with 5-s intervals also inhibited synaptic transmission by depletion of the RRP. Correlating with maintenance of healthy physiology, synaptic vesicle dynamics appeared to be normal, as demonstrated by presence of the first phase of EPSP slope during a single 50-Hz train, reflecting presence of an RRP resembling wild type (Fig. 4*D*). Additionally, the integrated EPSP slope averaged across multiple 50-Hz trains now resembled that of WT-SOD1-YFP synapses (*n* = 6 for each; Fig. 4*E*). Finally, in EM analyses (Fig. 4*F*), consistent with presence of a normal RRP, vesicles were observed abutting the AZ after iHFS was applied to a synapse infused with G85R-SOD1-YFP for 6 h. These results indicate that iHFS is able to preserve the RRP in the face of G85R-SOD1-YFP infusion. Interestingly, in G85R-SOD1-injected synapses (three out of five) with dramatic decreases in the slope of the PSP but remaining >50, application of one HFS train restored EPSP slope and the RRP size, without rescuing vesicle mobilization rate (Extended Data Fig. 4-1). However, in the other two synapses where PSP slopes dropped to below 50, adding one HFS further reduced them to almost 0. This suggests that to achieve the rescuing effects, the timing of the HFS application with regard to existing synaptic strength is important. However, the underlying molecular mechanism of this rescuing effect is unclear and requires further study. Altogether, the intermittent stimulation reverses toxicity of the misfolded protein by restoring synaptic vesicle availability at the AZ.

10.1523/ENEURO.0369-19.2020.f4-1 Extended Data Figure 4-1 Rescuing effects of HFS in dying synapses. In three out of five synapses where the EPSP slopes were significant inhibited by G85R-SOD1-YFP but were still above 50, one train of HFS surprisingly restored neurotransmission as indicated by the rescue of EPSP slope. Synaptic membrane potentials were measured in presynaptic and postsynaptic terminals before G85R-SOD1 infusion (***A***), after inhibition (***B***), and after one train of HFS (***C***). HFS partially restored the RRP size without rescuing vesicle mobilization rate in these synapses. In the other two synapses where EPSP slopes had dropped below 50, a train of HFS further decreased the EPSP slope to almost 0, consistent with its role in depleting RRP. Download Figure 4-1, TIF file.

### G85R-SOD1 induces Ca^2+^ increase in the presynaptic terminal

**Figure 5.**
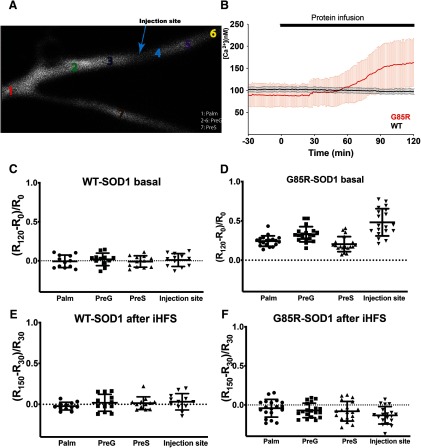
Presynaptic infusion of G85R-SOD1-His increased Ca^2+^ levels in the presynaptic terminal. Ratiometric live Ca^2+^ imaging was performed every 30 s after electrophoretic (100 nA current) infusion of a Ca^2+^ indicator, 1 mM fura-2 in 100 mM KCl, into the presynaptic axon through the first presynaptic stimulation electrode inserted into the palm (Fig. 1*A*). Fura-2 injection continued for 10 min, and the synapse was allowed to equilibrate for 30 min before SOD1 injection through the second electrode to ensure fura-2 was diffused evenly throughout the whole presynaptic terminal at roughly 100 μM. Ratiometric images were taken at Ex360 nm and Ex390 nm assisted by ultra-high-speed wavelength switching λ DG-4/5 xenon arc lamp system. Ratios at various locations (***A***) were calculated as (fluorescent intensity^360nm^)/(fluorescent intensity^390nm^) to provide a measure of intracellular Ca^2+^ concentrations. The blue arrow indicates the injection site for SOD1 proteins. 1: palm, 2–6: PreG, 4: infusion site, 7: PreS, an adjacent presynaptic axon branch which is smaller in size. ***B***, G85R-SOD1-His induced Ca^2+^ increases while WT-SOD1-His had no effect on Ca^2+^ concentration. Raw Ca^2+^ concentrations were derived from the equation [Ca^2+^] = Kd′(R−Rmin)/(Rmax−R) and averaged across 12 WT- and 19 G85R-SOD1-injected presynaptic terminals (PreG) respectively. Kd′, Rmin, and Rmax were calculated from standards as described in Materials and Methods. Although different synapses varied in their baseline Ca^2+^ concentrations, G85R consistently increased [Ca^2+^] at around 60 min after protein injection whereas WT had no effect. ***C***, ***D***, Baseline normalized Ca^2+^ ratio defined as (R_120_ – R_0_)/R_0_ (R_120_: 60 min after SOD1 injection, R_0_: before SOD1 injection) were plotted at four sites for WT (*n* = 12) and G85R (*n* = 19). G85R-SOD1 caused increases in [Ca^2+^] at all sites including the palm where Ca^2+^ channels are sparse or absent. The increase in [Ca^2+^] correlated roughly with G85R-SOD1 concentration, with the highest levels at the injection site and lowest in PreS, a smaller presynaptic axonal branch infused with fura-2 at a comparable concentration but with a lower SOD1 concentration due to slower diffusion from the injection site in PreG. ***E***, ***F***, iHFS applied at 30 min after SOD1 injection seemed to restore Ca^2+^ homeostasis in G85R-SOD1-injected synapses (*n *= 19), and this equilibrium lasted at least 2 h after iHFS, similar to that in WT (*n* = 12), this suggested the possibility that redistribution of Ca^2+^ is induced by iHFS. As expected, Ca^2+^ levels increased during HFS in synapses infused with either WT or G85R-SOD1 proteins (Extended Data Fig. 5-1).

Intrigued by the rescuing effect of iHFS in G85R-SOD1-infused synapses, we examined potential mechanisms. Since iHFS is known to alter Ca^2+^ dynamics, we wondered if aberrant Ca^2+^ signaling may underlie G85R-SOD1-YFP toxicity. To test this possibility, we performed ratiometric Ca^2+^ imaging using fura-2, a fluorescent Ca^2+^ indicator dye, which was delivered to the presynaptic axon electrophoretically and allowed to diffuse into the palm and presynaptic branches [Fig. 5*A*, regions of interest (ROIs) were selected to show 1: palm, 2–6: PreG, presynaptic axon which forms the giant synapse, and 7: PreS, another small presynaptic branch]. In parallel, the presynaptic axon was subjected to basal stimulation while both presynaptic and postsynaptic membrane potentials were recorded. The presence of fura-2 provides an indication of changes in Ca^2+^ levels and location during stimulation. To avoid interference with Ca^2+^ imaging, WT-SOD1 and G85R-SOD1 constructs used in these studies lacked the YFP tag and injections were monitored by co-perfusion of tetramethylrhodamine-dextran. While overall characteristics of synaptic function are similar with the introduction of fura-2, the impairment of synaptic transmission by G85R-SOD1 was delayed in the presence of fura-2 (>60 min, previously <30 min after G85R-SOD1 injection), probably due to the buffering of Ca^2+^ by indicator dye. This impairment was correlated with a concurrent rise in Ca^2+^ levels at 60 min. Synapses injected with fura-2 alone or tetramethylrhodamine-dextran alone kept constant EPSP normally for >2 h as those injected with WT. Furthermore, synapses injected with WT-SOD1 behaved similarly to those with WT-SOD1-YFP, excluding the potential role of YFP tag on synaptic transmission.

10.1523/ENEURO.0369-19.2020.f5-1 Extended Data Figure 5-1 Increases in Ca^2+^ levels under HFS. Synapses infused with WT-SOD1 (***A***) of G85R-SOD1 (***B***) exhibited increased Ca^2+^ levels upon HFS, mainly in the presynaptic terminals, with comparable changes at the protein infusion site and the rest of the terminal (PreG). The palm, which lacks Ca^2+^ channels did not show changes in Ca^2+^ influx, consistent with previous findings. G85R-SOD1 did not seem to alter the overall pattern, except for larger fluctuations particularly at the palm and the protein infusion site, which may indicate changes in Ca^2+^ homeostasis independent of Ca^2+^ channels. Download Figure 5-1, TIF file.

G85R-SOD1 infusion (*n* = 19) caused increases of Ca^2+^ throughout the presynaptic terminal compared with WT controls (*n* = 12), as derived from the ratios of (fluorescent intensity^360nm^)/(fluorescent intensity^390nm^) averaged across ROIs 2–6 (Fig. 5*B*). While WT-SOD1 under basal stimulation showed no change in Ca^2+^ concentration after 1 h protein infusion (Fig. 5*C*), G85R-SOD1 increased global Ca^2+^ concentration in the palm, the presynaptic giant axon (PreG), as well the other small presynaptic branch (PreS), with the most significant elevation at the site of G85R-SOD1 injection (Fig. 5*D*) perhaps due to the highest G85R-SOD1 concentration at the injection site. Both the palm and PreS regions, further away from the injection site, would contain lower concentrations of mutant protein due to diffusion. Interestingly, the palm which lacks plasma membrane Ca^2+^ channels and does not normally show Ca^2+^ increases during HFS did not exhibit increased Ca^2+^ with WT-SOD1 (Extended Data Fig. 5-1*A*), consistent with earlier findings (Smith et al., 1993). However, in the presence of G85R-SOD1, the palm also displayed aberrant Ca^2+^ increases with HFS (Extended Data Fig. 5-1*B*), suggesting that sources of Ca^2+^ other than plasma membrane Ca^2+^ channels may contribute to this Ca^2+^ misregulation. Taken together, the evidence suggests that G85R-SOD1-induced Ca^2+^ influx may mediate its inhibitory effects on synaptic transmission and the effect may depend on intracellular Ca^2+^ stores.

Given that iHFS rescued G85R-SOD1-associated synaptic defects, we wondered if iHFS could correct the aberrant localization of Ca^2+^ increases. Fura-2 ratiometric Ca^2+^ imaging demonstrated Ca^2+^ increases during HFS in both WT- and G85R-SOD1-injected synapses (Extended Data Fig. 5-1), confirming previous findings of HFS on Ca^2+^ dynamics by others (Smith et al., 1993). In the presence of G85R-SOD1, Ca^2+^ concentrations in the palm appeared more variable across the synapses, with some palm regions showing Ca^2+^ increases as high as those in the synaptic terminal, suggesting high baseline Ca^2+^ concentration. However, there seemed to be no significant difference between WT-SOD1-injected (*n* = 12) and G85R-SOD1-injected (*n* = 19) synapses at the injection site and the other locations within the presynaptic terminals during iHFS, indicating normal Ca^2+^ channel functions at the injection site, thereby excluding the possibility of physical membrane damage caused by the injection. Consistent with iHFS rescue in synaptic physiology and in contrast to G85R-SOD1-induced Ca^2+^ influx in the absence of iHFS, aberrant Ca^2+^ activation was not observed after iHFS in G85R-SOD1-injected synapses (Fig. 5*E*,*F*). These results suggest that iHFS may reset Ca^2+^ dynamics in the terminal and prevent the local Ca^2+^ overloads that contribute to synaptic dysfunction induced by G85R-SOD1.

### EGTA prevents toxic effects of G85R-SOD1-YFP on synaptic physiology and morphology

**Figure 6.**
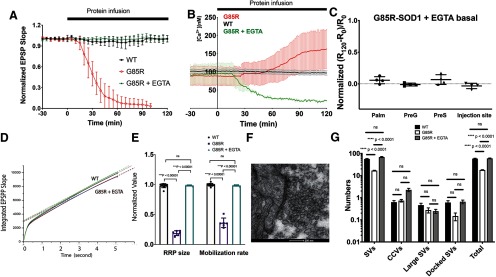
EGTA, a Ca^2+^ chelator, prevented G85R-SOD1-YFP-induced synaptic dysfunctions. ***A***, EPSP slopes of six double injected synapses (50 mM EGTA and 50 μM G85R-SOD1-YFP) were plotted from 30 min before the injection to 2 h after the injection. There was no reduction in EPSP slopes with EGTA and G85R-SOD1-YFP in contrast to that seen in synapses injected with G85R-SOD1-YFP alone (*n* = 8) or WT-SOD1-YFP alone (*n* = 5), data for G85R-SOD1 and WT-SOD1 from Figure 1*C* is included for comparison. All EPSP slopes were averaged and plotted with error bars indicating SE (for unnormalized data, see Extended Data Fig. 6-1). ***B***, Ratiometric fura-2 Ca^2+^ imaging showed comparisons of Ca^2+^ concentrations in the presynaptic terminals (PreG) injected with WT-SOD1, G85R-SOD1, and G85R-SOD1+ EGTA, EGTA prevented the Ca^2+^ increase caused by G85R-SOD1 (*n* = 4). Because of the chelating effect of EGTA, the basal level of Ca^2+^ decreased over time during co-infusion of G85R and EGTA. ***C***, Normalized ratios (subtracting levels with EGTA alone), positively correlated with Ca^2+^ concentrations, showed constant basal Ca^2+^ levels at various presynaptic regions injected with G85R-SOD1 in the presence of EGTA. ***D***, EPSP slopes from synapses injected with EGTA and G85R-SOD1 were integrated, normalized to the initial time point before the injection, and fit linearly to show RRP size as indicated by the intersection with the *y*-axis and the vesicle mobilization rate from RP to RRP as indicated by the slope of the linear fit. Compared with WT-SOD1-injected synapses (green) and synapses before injections (black), EGTA+G85R-SOD1-injected synapses showed little change in the RRP size and the mobilization 120 min after injection (red) and 180 min after injection (blue). ***E***, Quantification of RRP size and mobilization for synapses injected with WT-SOD1 (*n* = 11), or G85R-SOD1 (*n* = 6), or G85R-SOD1 + EGTA (*n* = 5) showed that EGTA prevented the decreases in RRP size and mobilization rate caused by G85R-SOD1-YFP. Error bars indicate SE. ***F***, Representative EM image showed abundant synaptic vesicles at the AZ of a synapse injected with EGTA and G85R-SOD1-YFP. ***G***, EGTA restored the synaptic vesicle numbers to normal at the AZs (102 AZs from three independent experiments for each group) ns: not significant.

The iHFS rescue experiments in combination with Ca^2+^ imaging and the delayed inhibition produced by fura-2 suggested that aberrant Ca^2+^ activation by G85R-SOD1 may be the cause of synaptic inhibition. To further test this hypothesis, we chelated free Ca^2+^ in the presynaptic terminal by co-injecting 50 mM EGTA (final concentration at 5–10 mM in the synapse). Previous studies had shown that EGTA levels <80 mM do not affect synaptic transmission (Adler et al., 1991). EGTA was sufficient to block the inhibitory effects of G85R-SOD1-YFP on synaptic transmission and G85R-SOD1-YFP-injected synapses maintained steady EPSPs for over 2 h without iHFS (*n* = 6), which was comparable to WT-SOD1-YFP-injected synapses (Fig. 6*A*; Extended Data Fig. 6-1; five WT-SOD1-infused and eight G85R-SOD1-infused synapses were also shown in Fig. 1*C*). Not surprisingly, ratiometric Ca^2+^ imaging using fura-2 did not show any local Ca^2+^ increases in synapses co-injected with G85R-SOD1 and EGTA (Fig. 6*B*,*C*). Defects in synaptic vesicle pools, number and distribution induced by G85R-SOD1 were also corrected by EGTA, as evidenced by the usual RRP size, and normal mobilization rate (Fig. 6*D*,*E*). Furthermore, EM showed comparable numbers of synaptic vesicles at the AZs (Fig. 6*F*). The numbers of docked and electron lucent synaptic vesicles were similar between the WT-SOD1 and G85R-SOD1+EGTA-infused synapses, while EGTA might slightly increase clathrin coating (Fig. 6*G*; 102 synapses for each group). These results suggest that Ca^2+^ dysregulation may underlie G85R-SOD1-associated synaptic dysfunctions and that limiting increases in free cytoplasmic Ca^2+^ with EGTA may restore normal synaptic morphology and function.

10.1523/ENEURO.0369-19.2020.f6-1 Extended Data Figure 6-1 Unnormalized EPSP slopes. EPSP slopes were measured and plotted without normalization for synapses infused with WT-SOD1-YFP (***A***), G85R-SOD1-YFP (***B***), and G85R-SOD1-YFP + EGTA (***C***). Download Figure 6-1, TIF file.

## Discussion

The discovery of giant fibers in the squid nervous system by J. Z. Young (Young and Keynes, 2005) >80 years ago led to several milestone discoveries in modern neuroscience, including the Nobel-prize-winning work on ionic mechanisms for initiating and propagating action potentials by Andrew Huxley and Alan Hodgkin (Schwiening, 2012). Although advanced imaging and genetic tools as well as new model systems have been developed in recent years, these classical mature synapse and axon models continue to be used to understand fundamental neurobiology and provide a unique model to study molecular mechanisms of human neurologic diseases. For example, the original discovery of the molecular motor kinesin in isolated axoplasm from the squid led to studies of axonal trafficking deficits resulting from altered motor function in several neurodegenerative diseases (Song et al., 2016; Brady and Morfini, 2017). In a similar way, studies of molecular mechanisms underlying basic synaptic physiology in the SGS have led to exploration of disease mechanisms involving synaptic dysfunction (Moreno et al., 2009). Here, we use the giant axo-axonic synapse in the squid stellate ganglion to study synaptic actions of mutant human SOD1 (G85R-SOD1) protein, which causes a familial form of ALS.

### Presynaptic injection of G85R SOD1 inhibits neurotransmission by disturbing synaptic vesicle physiology

Synaptic loss at affected neuromuscular junctions leading to a dying-back neuropathy appears to be a primary defect associated with pathology in ALS patients and mouse models bearing G85R-SOD1 mutations. Consistent with these observations, we found that infusion of ALS-associated human G85R mutant SOD1 protein but not the WT-SOD1 into the presynaptic terminal of the SGS significantly inhibited EPSPs, leading to a failure in eliciting postsynaptic action potentials. This occurred within 30 min of continuous infusion into the presynaptic terminal without diminishing the presynaptic action potentials, while infusion of the WT-SOD1 had no effect on synaptic transmission as evidenced by sustained firing of synapses with constant EPSPs (Fig. 1).

High-frequency stimulus protocols designed to deplete the RRP of presynaptic vesicles showed that vesicle dynamics were significantly inhibited by G85R-SOD1, reducing both the size of the RRP and the mobilization rate from the RP to the RRP (Fig. 2). This finding was further validated by EM data that demonstrated significant reductions of synaptic vesicles at AZs in the presence of G85R-SOD1 protein (Fig. 3). Ultrastructural studies provided additional evidence of altered vesicle trafficking as membranous structures resembling multivesicular bodies and autophagosomes were identified in presynaptic regions infused with G85R-SOD1, but not WT-SOD1. Inhibition of synaptic transmission was reversed by stopping presynaptic infusion of G85R-SOD1 and allowing sufficient time for the protein to diffuse out of the synapse *via* the presynaptic giant axon (Extended Data Fig. 1-2), which suggested that the synaptic machinery was not physically and permanently damaged.

### Nature of synaptic vesicle disturbance

A clue to the mechanism for G85R-SOD1-induced synaptic inhibition came from an experiment where the synapse was exposed to iHFS at 30-min intervals before and during G85R-SOD1 infusion. This was expected to inhibit synaptic transmission based on earlier results showing that continuous HFS depleted presynaptic RRP. Unexpectedly, G85R-SOD1 synapses exposed to iHFS maintained constant EPSPs for over 7 h, exhibiting normal synaptic vesicle dynamics and normal numbers of synaptic vesicles at AZs despite the presence of pathogenic SOD1 protein (Fig. 4). This finding led us to examine Ca^2+^ dynamics as HFS is known to redistribute Ca^2+^ both temporally and spatially (Smith et al., 1993).

Live cell ratiometric Ca^2+^ imaging studies showed that G85R-SOD1 consistently increased Ca^2+^ levels in presynaptic terminals with the largest increases at the injection site, the site with highest G85R-SOD1 concentration. Surprisingly, Ca^2+^ levels were also increased in the palm region, which lacks plasmalemmal Ca^2+^ channels and normally does not exhibit Ca^2+^ increases in response to HFS (Fig. 5). These data suggested that the source of Ca^2+^ may not be restricted to or dependent on local plasma membrane Ca^2+^ channels. This agrees with previous findings that Ca^2+^ influx solely through voltage-gated Ca^2+^ channels is not able to induce motor neuron death (Van den Bosch et al., 2002). Instead, release of Ca^2+^ from endoplasmic reticulum and/or mitochondrial stores is required, and these organelles may release Ca^2+^ in response to misfolded G85R-SOD1 protein.

### Co-injection of EGTA and application of iHFS prevent physiopathology

If increased free Ca^2+^ levels are responsible for synaptic failure, co-injection of EGTA, a Ca^2+^ chelator, at levels that do not affect synaptic transmission at the SGS should prevent synaptic inhibition by G85R-SOD1 by buffering free Ca^2+^. Consistent with the conclusion that presynaptic increases in free Ca^2+^ levels were responsible for the observed synaptic dysfunction, EGTA prevented inhibition of synaptic transmission when co-injected into the presynaptic terminal with G85R-SOD1, as documented by (1) EPSPs remained constant for over 2 h; (2) sufficient numbers of synaptic vesicle were present at the AZs with normal mobilization from RP to RRP; and (3) cytoplasmic free Ca^2+^ levels did not increase (Fig. 6). Similar effects were also achieved by iHFS applied every 30 min for over 7 h (Fig. 4). Interestingly, iHFS also abolished the Ca^2+^ increases induced by G85R-SOD1 (Fig. 5) perhaps by redistributing Ca^2+^ and restoring homeostasis. This process may alter regulation of other organelles such as ER, mitochondria, and autophagosomes in the synapse to restore overall synaptic health. However, determining the exact molecular mechanisms requires more in-depth studies.

Questions remain as to how increased cytoplasmic free Ca^2+^ alters synaptic vesicle dynamics in the presynaptic terminal. Ca^2+^ is a key player in many cellular and subcellular processes, is required for regular synaptic function, and is normally tightly regulated. In normal synapses, free Ca^2+^ increases are largely restricted to the AZ through local plasmalemmal channels during synaptic transmission (Sudhof, 2004). The levels of EGTA used in these studies do not affect Ca^2+^ influx at AZs but do buffer cytoplasmic free Ca^2+^. There are multiple Ca^2+^ sensors with a wide range of affinities in the presynaptic compartment, all of which may contribute to one or more of the complex steps involved in synaptic transmission and synaptic vesicle dynamics. While the role of some of these sensors is established, such as evidence that synaptotagmin is the Ca^2+^ sensor for synaptic vesicle fusion and neurotransmitter fast release, the physiological roles for many others remain unclear (Sudhof, 2004).

The existing literature has suggested a variety of potential mechanisms for Ca^2+^ related pathology in both human and mouse ALS-vulnerable neurons (Grosskreutz et al., 2010), such as the subgroups of motor neurons in spinal cord and brainstem expressing low levels of Ca^2+^ buffering proteins, even at the presymptomatic stages. Similarly, Ca^2+^ dysregulation has also been observed in several other neurodegenerative diseases and may lead to unfolded protein responses (UPRs) and ER stress-related signaling activation (Grosskreutz et al., 2010; Walker and Atkin, 2011; Hetz and Saxena, 2017; Remondelli and Renna, 2017), Ca^2+^ permeable AMPA receptor dysregulation (Tateno et al., 2004; Tortarolo et al., 2006; Van Den Bosch et al., 2006), ER/mitochondria membrane collapse (Tortarolo et al., 2006; Watanabe et al., 2016; Bernard-Marissal et al., 2018), and aberrant Ca^2+^ influx in affected neurons and glia. In addition, changes in protein expression in affected motor neurons (e.g., Ca^2+^ channels and regulators) may also contribute to dysregulation of Ca^2+^ levels in disease. While altered transcription may play a role in the slow progression of the disease, this is unlikely to contribute here given the time scale and reversibility of these effects as well as the lack of strong evidence of local transcription in the SGS.

In conclusion, our results in the SGS indicate that mutant SOD1 affects the regulation of free Ca^2+^ levels in the presynaptic terminal, leading to a disruption of normal vesicle trafficking. These effects were not restricted to regions near the AZs but were more extensive and affected regions of the presynaptic compartment that lack plasmalemmal Ca^2+^ channels. The effects of mutant SOD1 may occur without affecting the activity of local plasmalemmal Ca^2+^ channels required for neurotransmitter release at the AZ, given that (1) presynaptic regions lacking Ca^2+^ channels also showed increased levels of free Ca^2+^, (2) low levels of EGTA can reduce free Ca^2+^ levels in the presence of G85R-SOD1 without affecting Ca^2+^ influx through plasmalemmal Ca^2+^ channels responsible for normal synaptic transmission, and (3) the discovery that iHFS could rescue synaptic transmission. The effect of iHFS was unexpected and further argues for normal function of AZ Ca^2+^ channels in the plasmalemma independent of intracellular stores of Ca^2+^. The effect of iHFS suggests that further study is needed of how patterns of activity can preserve synaptic function and this may be a basis for novel therapeutic interventions in ALS.

The effects observed here may relate to those observed earlier in the squid giant axon innervating muscles in the mantle. There we found that G85R-SOD1-YFP inhibited axonal trafficking of presynaptic vesicles by activating a MAPK stress pathway, which impaired kinesin-based transport responsible for anterograde cargo transport (Song et al., 2013). In addition, we observed that G85R-SOD1 was associated with several synaptic proteins critical for synaptic transmission, such as synapsin and syntaxin-binding protein 5, at a much higher level than WT-SOD1-YFP. HSP110, an HSC70 co-chaperone which prevented the neurotoxicity of G85R-SOD1 in the axon by potentially sequestering or refolding G85R-SOD1, also abolished the association of Synapse-associated protein and syntaxin-binding protein 5 with G85R-SOD1-YFP (Song et al., 2013). The roles that G85R-SOD1 interactions with synaptic proteins may play in affecting synaptic vesicle dynamics as well as regulation of these interactions via Ca^2+^ dependent or independent pathways remain to be determined. Various neuropathogenic changes have been proposed to be a consequence of Ca^2+^ dysregulation, such as UPR and autophagy, in ALS and several other neurodegenerative diseases. Our EM data (Fig. 6) showed that EGTA, when co-injected with G85R-SDO1, restored synaptic morphology and prevented the formation of pre-autophagosome and adjoining pre-lysosomes, which were observed when presynaptic G85R-SOD1 was injected alone.

### Implication of acute toxic effects of G85R-SOD1 on SGS physiology as relates to longer term ALS pathology in mammalian synapses

Like many other adult-onset neurodegenerative diseases, one big puzzle in the field is “why does it take 40–50 years to develop ALS?” Several disease-related processes occur during aging which can be overcome in young neurons due to robust compensatory mechanisms. In this study, we focus on acute effects of pathogenic SOD1, but a number of age-related factors may explain the delayed onset of clinical symptoms.

Unlike the acute injection of pathogenic SOD1 in this study where we observed the presence of pre-autophagosome and pre-lysosome formation within the time frame of both electrophysiological recordings and morphologic studies, ALS-associated mutant SOD1 in patients misfolds and accumulates gradually to pathologic levels over time, allowing adaptive changes in cellular and molecular mechanisms underlying synaptic transmission. This is due to, at least partially, mechanisms for actively removing pathogenic proteins in early stages of disease, which may be sufficient to slow the accumulation of toxic materials. However, these clearance mechanisms may eventually be unable to keep up with misfolded protein accumulations. By directly injecting defined levels of mutant SOD1 into an isolated SGS system, we ensured accumulation of misfolded protein locally in the synapses within a short period of time which limits compensatory effects in the isolated synapse with little protein degradation and synthesis in this time frame.

Similar to robust protein homeostatic pathways during development and maturation, Ca^2+^ buffering mechanisms and synaptic transmission may be redundant early on in the complex system such as the mammalian models where neuronal circuits (including the other cell types) are intact and dynamic. They can adapt to slowly increased stresses created by the presence of mutant SOD1, much as synaptic functions exhibit adaptive changes during normal aging. The use of isolated acute preparations of giant synapse also eliminates the possibility that transcriptional changes of synaptic proteins can compensate for the effects of toxic proteins on synaptic transmission machinery. For example, the question of whether ALS-affected neurons show hyper- or hypo- excitability has been debated for some time. As a matter of fact, several groups have reported hyperexcitability of motor neurons at early stages followed by hypoexcitability at later stages of ALS in both iPSC culture models and in mouse models. These changes may be due to various compensatory mechanisms at the molecular, cellular and systemic levels which also may contribute to the delayed onset of symptoms. Here, we can isolate inhibitory effects due to the simplicity of the squid synapse model system where many other factors, such as compensation from other neurons, changes in gene expression or axonal transport, or the impact of a complex environment can no longer play a role.

Furthermore, it is worth keeping in mind that synaptic loss, as a result of gradual decrease in synaptic strength as demonstrated here, occurs long before cell death and can be observed in presymptomatic stages of both ALS mice and patients. By measuring EPSP slopes and vesicle dynamics in the SGS, we have provided a sensitive readout of neurotransmission in real time and revealed changes in synaptic strength which are more subtle in pre-symptomatic patients and mouse models but are detectable long before clinically relevant behavioral abnormalities appear. Correlating Ca^2+^ dynamics and HFS effects with synaptic health in real time illuminates the kinetic changes in the presynaptic terminal with high temporal and spatial resolution, which suggests the importance of identifying potential therapeutic time windows. The acute effects demonstrated here may illustrate molecular mechanisms underlying disease pathology that are manifested over time in patients.

Relevant to mammalian physiology and pathology, the SGS also shares fundamental synaptic machineries with neuromuscular junctions in human, making it a unique ALS-disease model with a potential for answering important but otherwise difficult questions about pathogenic mechanisms and synaptic transmission in motor neuron diseases. The SGS provides a unique platform for (1) performing live measurements of both presynaptic and postsynaptic activities in response to disease-associated proteins with temporal and spatial resolutions surpassing those in most other model systems; (2) conducting morphologic studies (e.g. EM) in the same preparations characterized by electrophysiological studies and live-imaging of cellular and molecular pathways; (3) evaluating pharmacological inhibitors, biochemical tools, and electrophysiological protocols that may rescue disease phenotypes in a time dependent manner; and (4) identifying translatable mechanistic findings, due to the highly conserved synaptic machinery, that will provide better understanding and treatment of multiple neurodegenerative diseases where misfolded proteins cause disease pathology.

In conclusion, the development of novel models for study of neurodegenerative diseases such as those using the squid nervous system has the potential to provide unique insights into disease mechanisms. For example, the combination of our electrophysiological studies in the giant synapse with studies of axonal transport in isolated axoplasm from the postsynaptic giant axon of the squid, allowed us to identify signaling pathways altered by these pathogenic proteins (Song et al., 2013). These results were then developed further in mouse models of ALS and patient tissues or iPSCs. Our results here are also consistent with previous findings of Ca^2+^ dysregulation and synaptic dysfunction in both ALS mice and patient derived neurons (Grosskreutz et al., 2010; Tradewell et al., 2011; Leal and Gomes, 2015). For example, affected neurons exhibit aberrant Ca^2+^ dynamics, which leads to hyperexcitability or hypoexcitability at various stages (Wainger et al., 2014; Devlin et al., 2015; Martínez-Silva et al., 2018). Further, oculomotor neurons, which are relatively spared in ALS, have a higher buffering capacity for Ca^2+^ (Vanselow and Keller, 2000; Obál et al., 2006), which was thought to contribute to their overall resistance to pathology in patients and mouse models. Those data were unclear, however, as to whether these changes involved plasmalemmal Ca^2+^ channels or intracellular Ca^2+^ stores or both, an issue that could be addressed in the SGS.

Given recent work on the squid genome and improved mariculture protocols that have the potential to allow systematic long-term studies of squid nervous systems, there will be opportunities to develop novel transgenic ALS squid models. Therefore, studies using the SGS will continue to complement studies using human iPSCs and animal models of ALS as we seek to understand how multiple disease related pathways can independently affect the pleiotropic neuronal functions that interact to produce the phenotype of ALS, a multifactorial disease with a complex disease pathology.

## Synthesis

Reviewing Editor: Christian Hansel, University of Chicago Decisions are customarily a result of the Reviewing Editor and the peer reviewers coming together and discussing their recommendations until a consensus is reached. When revisions are invited, a fact-based synthesis statement explaining their decision and outlining what is needed to prepare a revision will be listed below. The following reviewer(s) agreed to reveal their identity: NONE.

This paper describes the acute effects of the ALS mutant G85R on at synapse. Dysregulation of calcium and decreased synaptic transmission can be rescued (or avoided) by a calcium chelator or by stimulation paradigms. The findings are intriguing and of interest with regards to the basic biology underlying ALS. Gain of function mutations in the SOD1 protein leads to ALS. Altered synaptic function may play a role in the disease. Although there are both sporadic and inherited (10% of cases) forms of ALS, the clinical symptoms are similar leading to the possibility that all forms lead to a common (similar) pathway. In this study, the authors make a good case that mutant SOD1 produces a dysregulation of intracellular calcium which leads to alterations in synaptic vesicle availability and subsequent synaptic transmission. The reviewers found the paper to be interesting. The squid giant synapse represents a good model system.

The revised version contains many necessary corrections. Noted are the scatter plots, better attention to statistics, better context in the discussion (but it is dragging a bit now) and addition of primary data. We were specially intrigued by the rescue effect in Figure 4-1. It thus appears that HFS not only prevents synaptic weakening (4C), it can also rescue it (4-1)! Despite these improvements, the paper in its present form still contains aspects that should be addressed.   1)Statistics of EM data. The authors now clarify that 5 individual giant synapses were analyzed. They also state that 102 synaptic profiles were counted. It is unclear whether this is per giant synapse or overall. In either case, the appropriate statistical n is NOT the number of synaptic profiles, but the number of biological replicates, ie in this case 5 as stated in the text or 3 as stated in the rebuttal. The authors could do a nested ANOVA or indicate in the scatter plot the 5 (or 3) replicates by different symbols. Panels C and F of Fig3 certainly make it look as if there are different populations.  Panel 3D and 3E show the limits of a simple scatter plot. At first, it appears that horizontal lines were inserted, rather than overlapping symbols. Small vertical offsets or a violin plot might help.   2)Confusion about excitability and evoked synaptic transmission. As pointed out for the previous version, it is irrelevant for this investigation whether the postsynaptic axon fires an action potential or not. What matters is how strong synaptic transmission is. The EPSP slope, if measured early, gives a good correlate of the amount of transmitter that was released. The confusion between postsynaptic AP and release is evident throughout the paper, but most obvious in Fig2 and Fig4-1. This is not a trivial semantic point, but a fundamental concept of synaptic physiology that the authors need to address.   3)Figure 2. Panels A-C do occupy space without conveying relevant information on neurotransmitter release. Figure 1-1, especially panel A, indicates one way on how to illustrate the raw data.  Fig 2 panel D shows the slopes for the first HFS train and Fig 2-1 shows the 6th train, illustrating the strong decrement in synaptic transmission. It would be interesting to plot the slope of the first and the last EPSP for all six trains in WT and G85R. It would illustrate how much ‘recovery’ happens in the five seconds between stimuli. It might also guide the reader through this unusual dataset.   4)New data. The new data showing that some of the SOD1 injected effects are reversible by HFS is highly intriguing and increases the impact of the study. Unfortunately, this data is relegated to ‘extended data’ and not analyzed in any detail. Again, the text and figure legends refer to postsynaptic firing. The authors state that in 3 out of 5 experiments “...where postsynaptic action potentials were no longer elicited, application of one HFS train restored action potential and the RRP size...” (lines 282-4). First, the authors must state and illustrate in Fig 4-1 what happened in the 2 other experiments. Second, it is not the presence of absence of a postsynaptic AP that matters, it is neurotransmitter release (see above). Could it be that limited rescue was observed in all five experiments?

  5). The time course of the result raises questions. We were hoping for a thoughtful discussion of how an effect that was observed in 30 minutes in squid giant synapse could translate to a human disease that manifests over decades. Instead the authors write (on page 20): “The squid model has several advantages. First, we “shortened” the disease progression by directly injecting misfolded mutant SOD1 into the presynaptic terminal”.
