# Supplementary figures and images for: Synaptic Actions of Amyotrophic Lateral Sclerosis-Associated G85R-SOD1 in the Squid Giant Synapse
Source: eNeuro. 2020 Apr 9;7(2):ENEURO.0369-19.2020. doi: 10.1523/ENEURO.0369-19.2020 (PMC7177748; doi:10.1523/ENEURO.0369-19.2020)

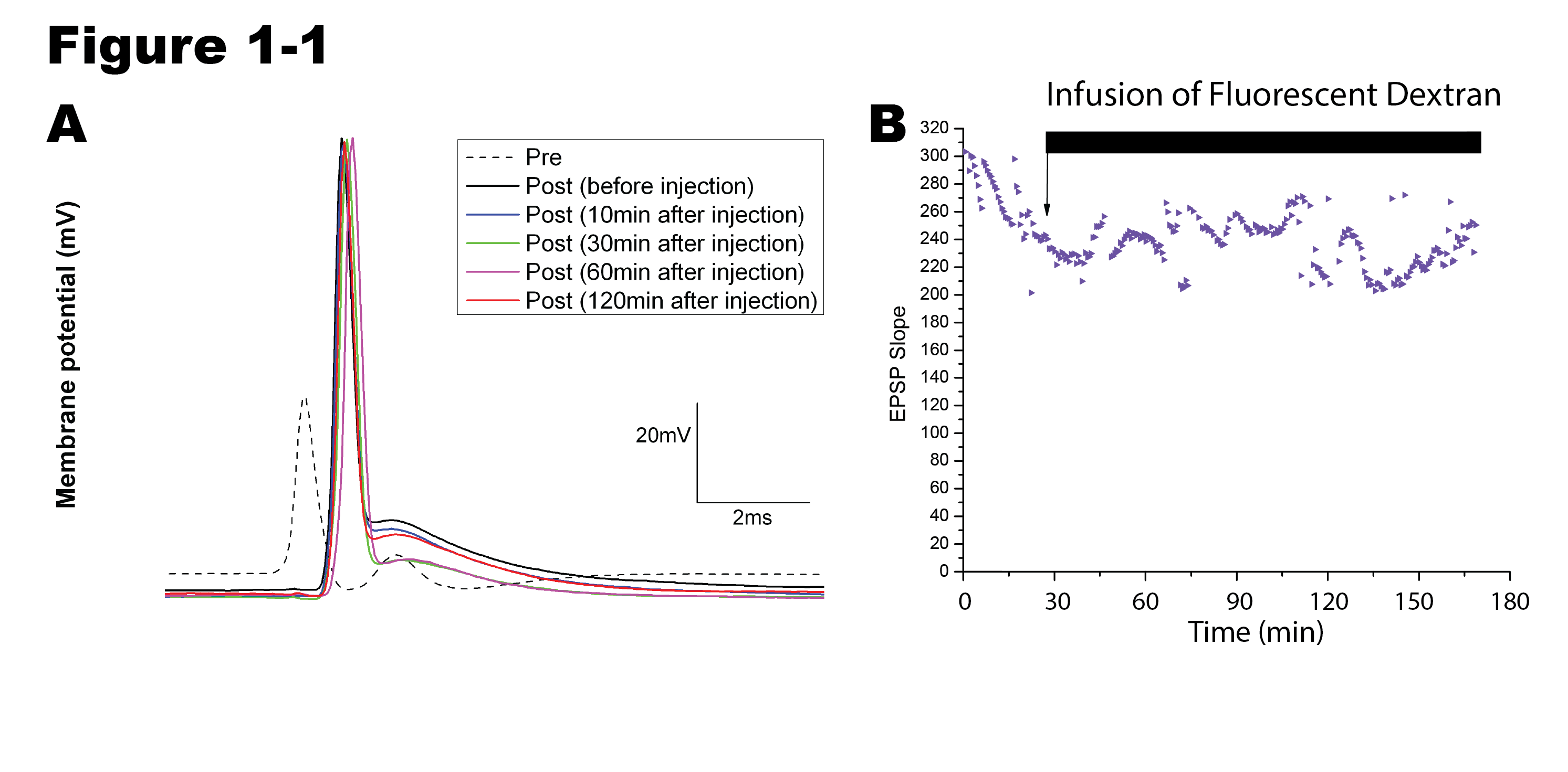

Supplement: Extended Data Figure 1-1. — No effects of fluorescent dextran on synaptic transmission. Synapses infused with rhodamine-dextran alone kept firing for >2 h, without significant changes in synaptic strength as evidenced by constant postsynaptic membrane potential waveform (A) and EPSP slope (B). Download Figure 1-1, TIF file. [file enu-eN-NWR-0369-19-s01.tif]

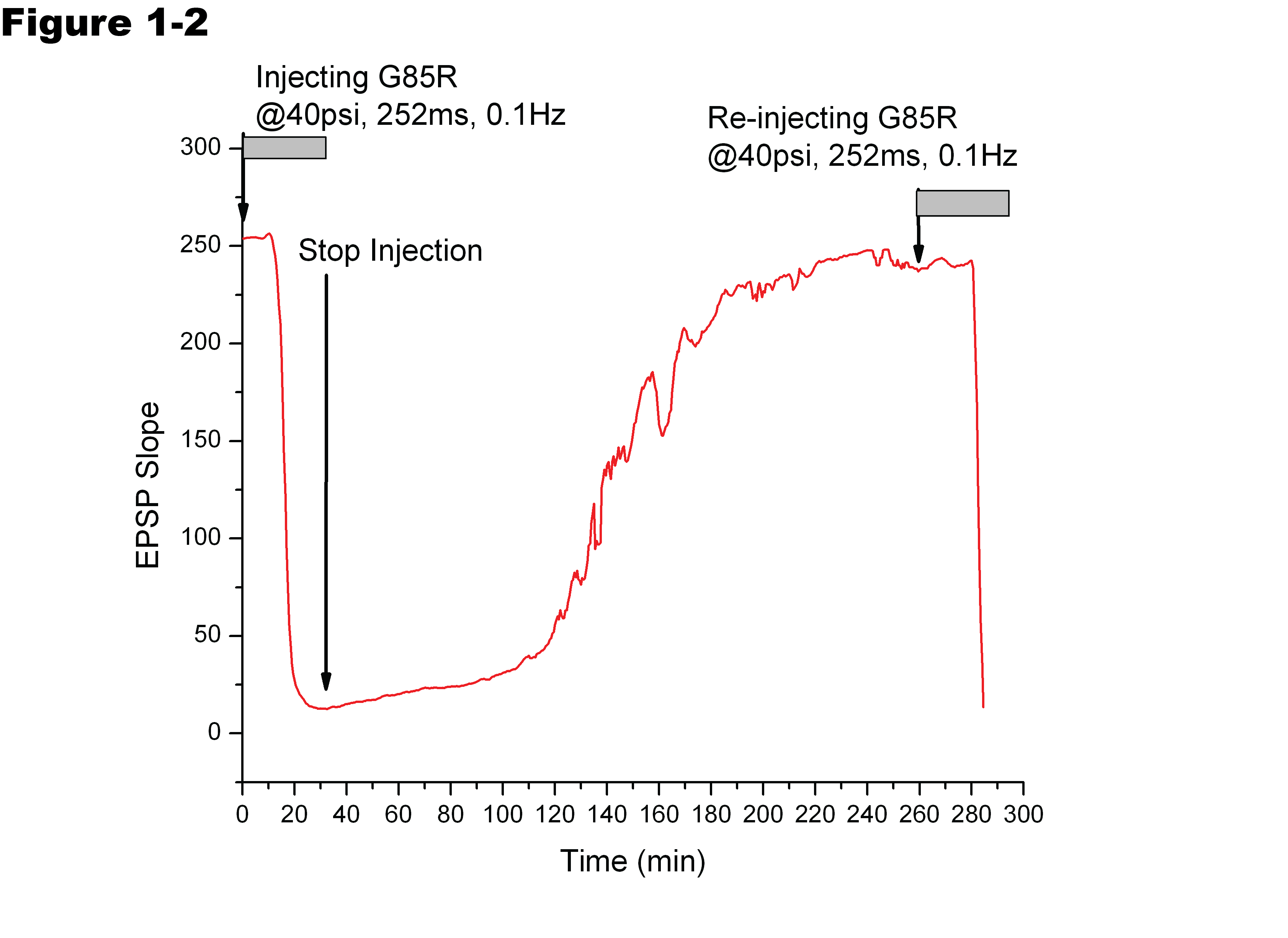

Supplement: Extended Data Figure 1-2 — Recovery of synaptic transmission after the removal of G85R-SOD1-YFP. The synapse was injected with G85R-SOD1-YFP continuously for 30 min until the significant reduction in EPSP slope and the failure to elicit a postsynaptic action potential. As the injection stopped, G85R-SOD1-YFP slowly diffused away from the presynaptic terminal to the axon, leading to a full recovery of EPSP after 150 min, suggesting that synaptic machinery remained intact after treatment. After the synapse and neurotransmission were stabilized for more than 1 h, G85R-SOD1-YFP was injected again and a similar inhibitory effect was observed. Download Figure 1-2, TIF file. [file enu-eN-NWR-0369-19-s02.tif]

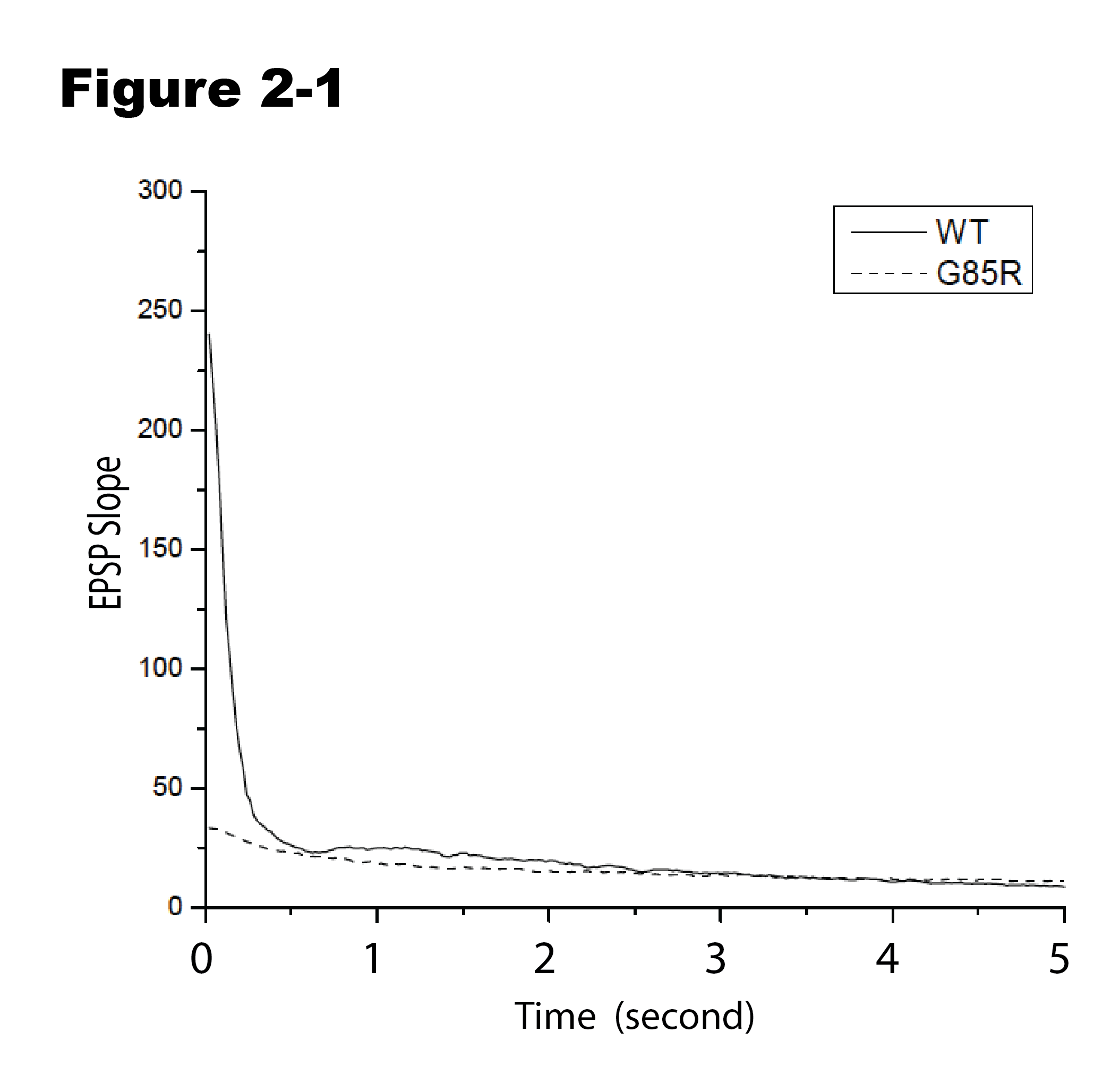

Supplement: Extended Data Figure 2-1 — Significant inhibition of EPSP by continuous HFS in G85R-SOD1-YFP-infused synapse. EPSP slopes taken during the 6th train in a set of continuous trains of HFS showed even more dramatic decreases by G85R-SOD1-YFP, compared with the WT-SOD1-YFP, particularly at the beginning, suggesting limited synaptic vesicle availability, consistent with the morphological changes found in EM. Note that in panel A, the EPSP slope with G85R-SOD1-YFP in the 1st train was initially >150, while in the 6th train, it never got above 40. In contrast, the initial EPSP slopes in the 1st train and the 6th train were almost identical after perfusion with WT-SOD1-YFP. Download Figure 2-1, TIF file. [file enu-eN-NWR-0369-19-s03.tif]

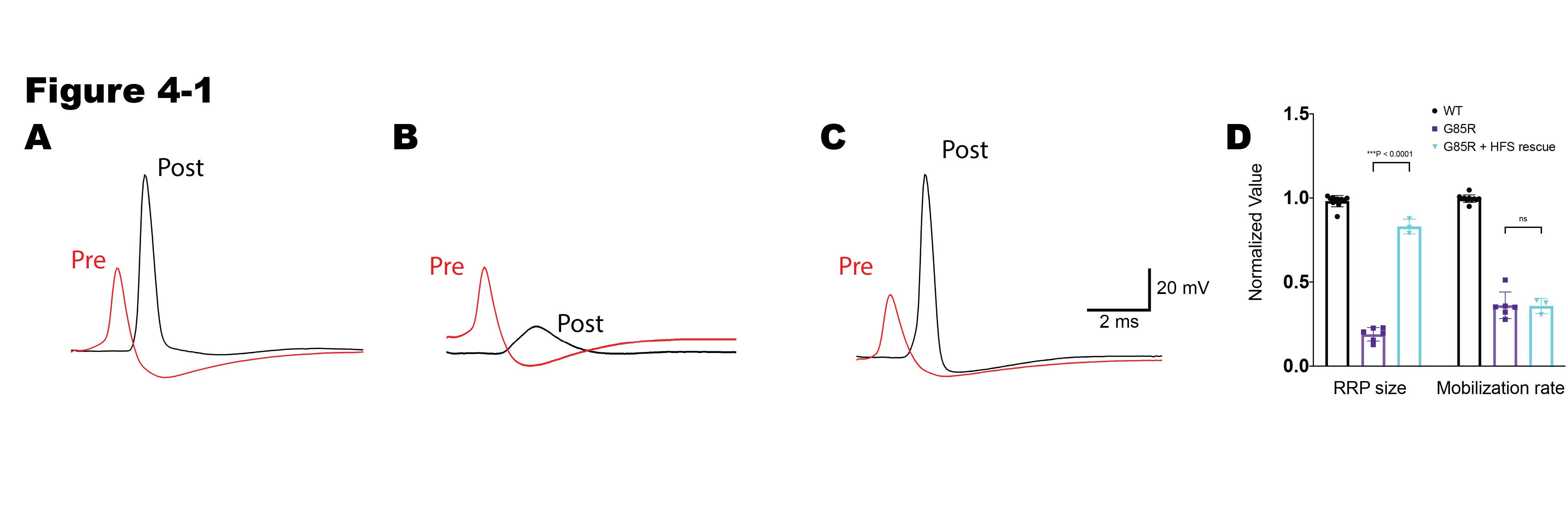

Supplement: Extended Data Figure 4-1 — Rescuing effects of HFS in dying synapses. In three out of five synapses where the EPSP slopes were significant inhibited by G85R-SOD1-YFP but were still above 50, one train of HFS surprisingly restored neurotransmission as indicated by the rescue of EPSP slope. Synaptic membrane potentials were measured in presynaptic and postsynaptic terminals before G85R-SOD1 infusion (A), after inhibition (B), and after one train of HFS (C). HFS partially restored the RRP size without rescuing vesicle mobilization rate in these synapses. In the other two synapses where EPSP slopes had dropped below 50, a train of HFS further decreased the EPSP slope to almost 0, consistent with its role in depleting RRP. Download Figure 4-1, TIF file. [file enu-eN-NWR-0369-19-s04.tif]

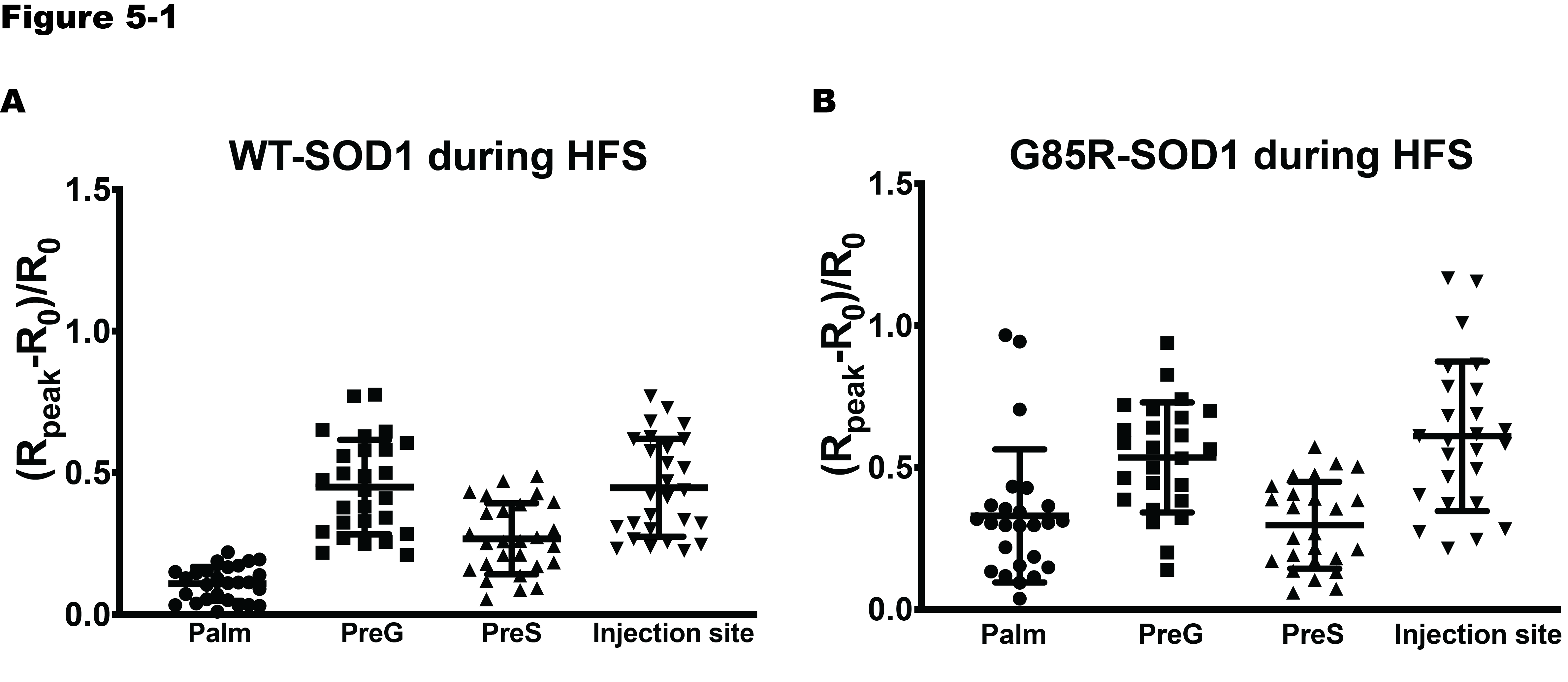

Supplement: Extended Data Figure 5-1 — Increases in Ca2+ levels under HFS. Synapses infused with WT-SOD1 (A) of G85R-SOD1 (B) exhibited increased Ca2+ levels upon HFS, mainly in the presynaptic terminals, with comparable changes at the protein infusion site and the rest of the terminal (PreG). The palm, which lacks Ca2+ channels did not show changes in Ca2+ influx, consistent with previous findings. G85R-SOD1 did not seem to alter the overall pattern, except for larger fluctuations particularly at the palm and the protein infusion site, which may indicate changes in Ca2+ homeostasis independent of Ca2+ channels. Download Figure 5-1, TIF file. [file enu-eN-NWR-0369-19-s05.tif]

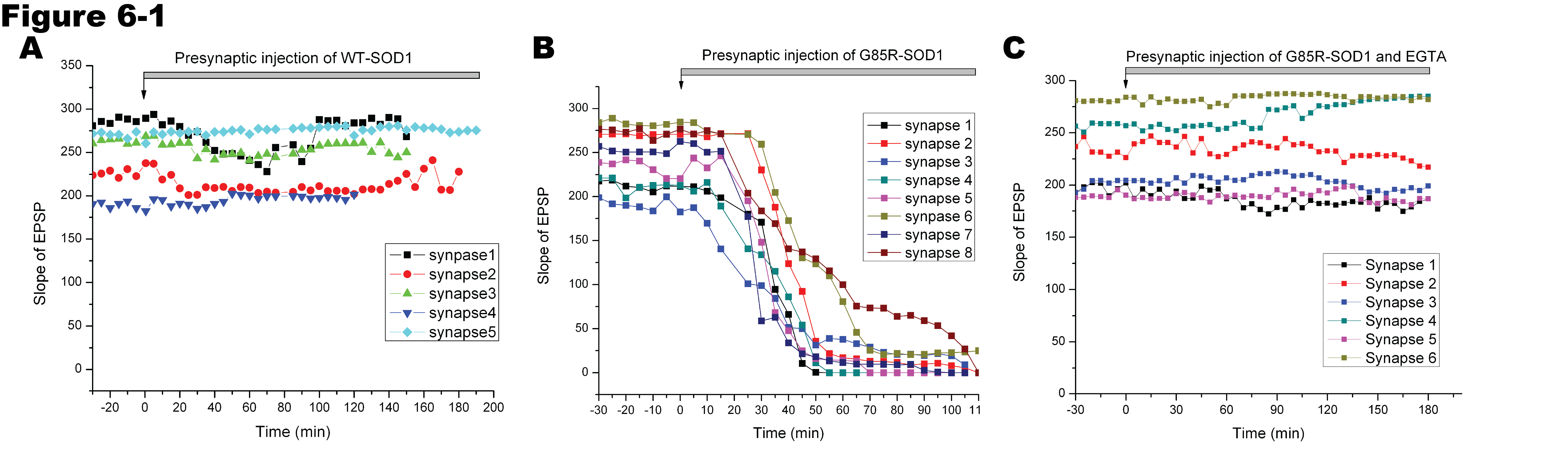

Supplement: Extended Data Figure 6-1 — Unnormalized EPSP slopes. EPSP slopes were measured and plotted without normalization for synapses infused with WT-SOD1-YFP (A), G85R-SOD1-YFP (B), and G85R-SOD1-YFP + EGTA (C). Download Figure 6-1, TIF file. [file enu-eN-NWR-0369-19-s06.tif]
